# Kirschner Wire Breakage during Removal Requiring Retrieval

**DOI:** 10.1155/2016/7515760

**Published:** 2016-10-24

**Authors:** Kai Yuen Wong, Rosalind Mole, Patrick Gillespie

**Affiliations:** ^1^Salisbury NHS Foundation Trust, Salisbury SP2 8BJ, UK; ^2^Cambridge University Hospitals NHS Foundation Trust, Cambridge CB2 0QQ, UK

## Abstract

Kirschner wires (K-wires) are widely used for fixation of fractures and dislocations in the hand as they are readily available, reliable, and cost-effective. Complication rates of up to 18% have been reported. However, K-wire breakage during removal is rare. We present one such case illustrating a simple technique for retrieval. A 35-year-old male presented with a distal phalanx fracture of his right middle finger. This open fracture was treated with K-wire fixation. Postoperatively, he developed a pin site infection with associated finger swelling. The K-wire broke during removal with the proximal piece completely retained in his middle phalanx. To minimise risk of osteomyelitis, the K-wire was removed with a novel surgical technique. He had full return of hand function. Intraoperative K-wire breakage has a reported rate of 0.1%. In our case, there was no obvious cause of breakage and the patient denied postoperative trauma. On the other hand, pin site infections are much more common with reported rates of up to 7% in the hand or wrist. K-wire fixation is a simple method for bony stabilisation but can be a demanding procedure with complications often overlooked. It is important to be aware of the potential sequelae.

## 1. Introduction

Kirschner wires (K-wires) are widely used for fixation of fractures and dislocations in the hand as they are readily available, reliable, and cost-effective [1]. Complications have been reported in up to 18% of such cases including infection, pin loosening, loss of reduction, and pin migration [1, 2]. K-wire breakage during removal is rare with one reported case in the literature [3]. We present a case of a retained broken K-wire, which required retrieval due to infection. A 35-year-old man sustained an oblique, displaced fracture to his distal phalanx which was managed with Kirschner wire (K-wire) fixation. Four weeks postoperatively, he developed a pin site infection and, during the removal, the K wire broke. This case report describes a novel technique devised to retrieve the retained K-wire. A review of the literature demonstrates that K-wire complications occur relatively infrequently but being aware of possible management options should they do is important.

## 2. Case Presentation

**Figure 1:**
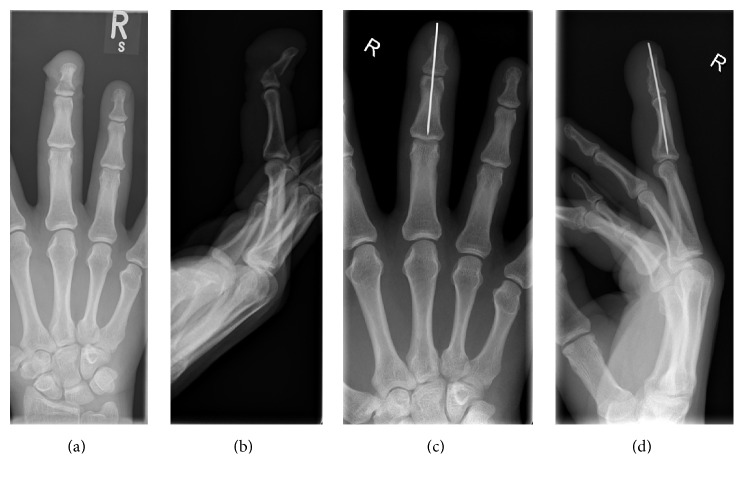
X-rays showing displaced oblique fracture of right middle finger distal phalanx (a, b), which was treated with Kirschner wire fixation (c, d).

**Figure 2:**
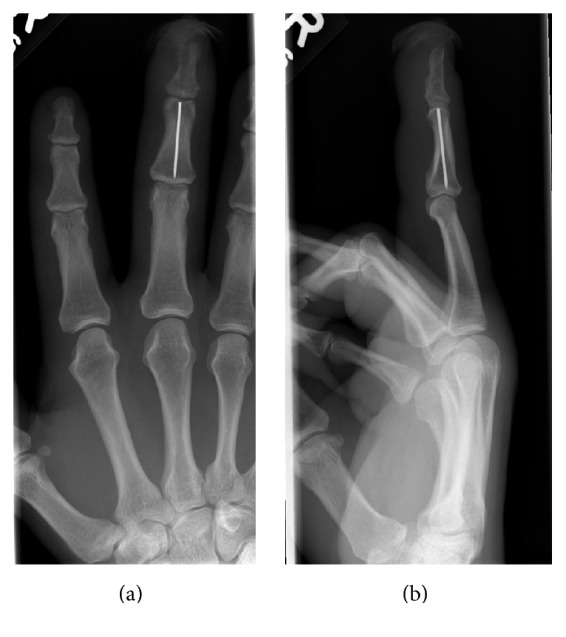
X-rays showing retained Kirschner wire in the right middle finger phalanx.

**Figure 3:**
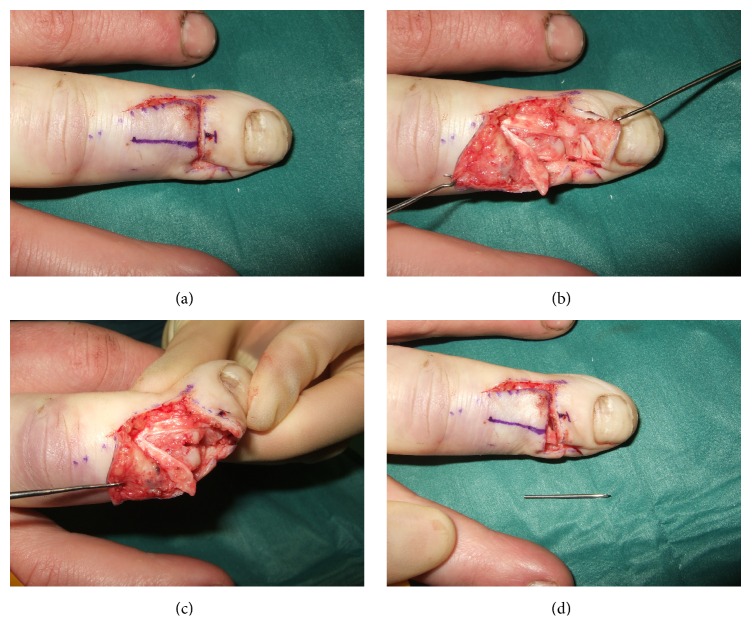
Right middle finger skin incision (a) with central line marking K-wire position based on fluoroscopy. Extensor tendon divided on ulnar side to access distal interphalangeal joint (b). Incision made dorsally on middle phalanx down to K-wire allowing it to be pushed out distally, facilitated by radial deviation at distal interphalangeal joint (c-d).

A 35-year-old office worker trapped his nondominant right middle finger in a door. X-ray showed a displaced distal phalanx oblique shaft fracture (Figures 1(a) and 1(b)). This open fracture was treated with debridement and K-wire fixation (Figures 1(c) and 1(d)). Four weeks postoperatively, he developed a pin site infection with associated finger swelling. This resolved with antibiotics but the K-wire broke during removal with the proximal piece completely retained in his middle phalanx (Figure 2). Due to risk of osteomyelitis from the pin site infection, the retained K-wire required removal. The patient went to theatre for removal of the K-wire under local anaesthesia (Figure 3). As the retained wire was located within the middle phalanx, access to the channel of insertion was technically challenging. A novel technique was devised to overcome this. Access to the distal interphalangeal joint (Figure 3(a)) was gained by dividing 40% of the extensor tendon on the ulnar side (Figure 3(b)). An incision was made on the dorsal surface of the middle phalanx straight down to the K-wire to allow it to be pushed out distally. The latter was facilitated by radial deviation at the distal interphalangeal joint (Figures 3(c) and 3(d)). Following washout of the wound, the extensor tendon was repaired to periosteum and the other slip with 5/0 nylon. He had an uneventful recovery; at one year, the DIP joint function was near normal with normal hand function [4].

## 3. Discussion

K-wire fixation is common and its rigidity is effective if not better than other modalities of fixation [5]. It is therefore not surprising that K-wire breakage is rare. In a prospective review of 11,856 orthopaedic procedures to determine the frequency of intraoperative instrument breakage, Pichler et al. reported K-wire breakage in only 14 cases (0.1%) [6]. In our patient, the cause of K-wire breakage is not obvious. The patient denied postoperative trauma or attempts to mobilise his distal interphalangeal joint.

On the other hand, pin site infections are much more common. In a series of 137 patients requiring K-wire fixation in the hand or wrist, Botte et al. observed K-wire related infection in 10 patients (7%) including osteomyelitis in 2 patients (1%) [2]. Similarly, Stahl and Schwartz reported a K-wire complication rate of 15% in a series of 236 patients, with 13 patients developing pin site infection (6%) and 1 case of osteomyelitis (0.4%) [1].

## References

[1] Stahl S., Schwartz O. (2001). Complications of K-wire fixation of fractures and dislocations in the hand and wrist. *Archives of Orthopaedic and Trauma Surgery*.

[2] Botte M. J., Davis J. L. W., Rose B. A. (1992). Complications of smooth pin fixation of fractures and dislocations in the hand and wrist. *Clinical Orthopaedics and Related Research*.

[3] Hong S. J., Lee H. J., Kim J. Y., Eo S. R., Cho S. H. (2013). K-Wire breakage during metalware removal due to a defective K-wire shaft. *Archives of Plastic Surgery*.

[4] Wong K. Y., Mole R., Gillespie P. (2016). Kirschner wire breakage during removal. *The British Journal of Surgery Society*.

[5] Fyfe I. S., Mason S. (1979). The mechanical stability of internal fixation of fractured phalanges. *Hand*.

[6] Pichler W., Mazzurana P., Clement H., Grechenig S., Mauschitz R., Grechenig W. (2008). Frequency of instrument breakage during orthopaedic procedures and its effects on patients. *Journal of Bone and Joint Surgery A*.

